# The complexity of diagnosis of FMF in children living outside typical of this disease in the region

**DOI:** 10.1186/1546-0096-12-S1-P274

**Published:** 2014-09-17

**Authors:** Yury M Spivakovskiy, Olga Skupova, Yuri Chernenkov, Anastasia Kadura, Anna Spivakovskaya, Elena Shulgina

**Affiliations:** 1 Department of Hospitality Pediatrics, Saratov State Medical University, Saratov, Russian Federation

## Introduction

Currently diseases from the group autoinflammatory syndromes pays much attention in the literature and real clinical practice. Among the diseases in this group is the most common Familial Mediterranean Fever (FMF). For this disease characterized by loss of patients of certain national groups, and also characterized by a specific geographical area of distribution. However, due to the growth of migration and assimilation processes, patients with this disease are increasingly can be detected outside the historically significant areas. In the absence of constant alertness doctors errors may occur in the diagnosis of this condition.

## Objectives

To evaluate the clinical features of and difficulties in diagnosis of in children living outside typical for this disease territory.

## Methods

The study was a retrospective study of all patients with FMF followed at the clinic of Hospital Pediatrics Saratov State Medical University named V.I. Razumovsky from September 2008 to January 2014. We reviewed the charts of 5 patients with FMF diagnosed according to the criteria of Tel-Hashomer that, however, adapted for populations with high some of the frequency of occurrence of this syndrome.

## Results

Children with newly diagnosed FMF were aged 1 year 11 months to 17 years. National of 4 children - Armenians, 1 child - Greek. The onset of the disease is marked in the age of 4 years (8 months. - 4 years). All children until this has been observed with different diagnoses, reflecting the assumed acute inflammatory disease (pneumonia, and so on). Suspicion for the presence auto inflammatory syndrome is not expressed. All children showed mixed option FMF: in five cases were abdominal - fever syndrome, in 2 cases - articular syndrome in 2 cases - thoracic syndrome. In the General analysis of the blood - accelerated ESR up to 25-50 mm/h. All patients on the background of fever have been reported increase of CRP to high numbers to the rapid decline after knocking febrile syndrome. In none of the cases are not recorded signs of amyloidosis and adhesive disease. Draws attention to the fact that younger children noted the minimum number of criteria Tel- Hashomer. In 2 cases of large criteria was not revealed. However, the detection of even a small number of small or supporting criteria FMF amid careful exception important foci of infection in conjunction with national identity are a pretext to perform a genetic examination. In all 5 cases at the final stage of the survey were identified mutations characteristic of the FMF - mutations M694V and V726A in compound heterozygous condition; in 2 cases - mutation M694V in the homozygous state; in one case detected strengthening electrophoretic mobility exon 10 MEFV gene, which is located more than 90% of mutations that are logged when FMF, corresponding mutation M694V in the heterozygous state.

## Conclusion

In pediatric practice even one symptom of recurrent fever combined with belonging to the respective ethnic group should be grounds for exclusion of FMF.

## Disclosure of interest

None declared.

